# Optimal Time Intervals in Two-Stage Takeover Warning Systems With Insight Into the Drivers’ Neuroticism Personality

**DOI:** 10.3389/fpsyg.2021.601536

**Published:** 2021-03-08

**Authors:** Wei Zhang, Yilin Zeng, Zhen Yang, Chunyan Kang, Changxu Wu, Jinlei Shi, Shu Ma, Hongting Li

**Affiliations:** ^1^Department of Psychology, Zhejiang Sci-Tech University, Hangzhou, China; ^2^Department of Industrial Engineering, Tsinghua University, Beijing, China; ^3^Modern Industrial Design Institute, Zhejiang University, Hangzhou, China

**Keywords:** takeover process, time intervals, neuroticism, situation awareness, takeover performance, lead time

## Abstract

Conditional automated driving [level 3, Society of Automotive Engineers (SAE)] requires drivers to take over the vehicle when an automated system’s failure occurs or is about to leave its operational design domain. Two-stage warning systems, which warn drivers in two steps, can be a promising method to guide drivers in preparing for the takeover. However, the proper time intervals of two-stage warning systems that allow drivers with different personalities to prepare for the takeover remain unclear. This study explored the optimal time intervals of two-stage warning systems with insights into the drivers’ neuroticism personality. A total of 32 drivers were distributed into two groups according to their self-ratings in neuroticism (high vs. low). Each driver experienced takeover under the two-stage warning systems with four time intervals (i.e., 3, 5, 7, and 9 s). The takeover performance (i.e., hands-on-steering-wheel time, takeover time, and maximum resulting acceleration) and subjective opinions (i.e., appropriateness and usefulness) for time intervals and situation awareness (SA) were recorded. The results showed that drivers in the 5-s time interval had the best takeover preparation (fast hands-on steering wheel responses and sufficient SA). Furthermore, both the 5- and 7-s time intervals resulted in more rapid takeover reactions and were rated more appropriate and useful than the 3- and 9-s time intervals. In terms of personality, drivers with high neuroticism tended to take over immediately after receiving takeover messages, at the cost of SA deficiency. In contrast, drivers with low neuroticism responded safely by judging whether they gained enough SA. We concluded that the 5-s time interval was optimal for drivers in two-stage takeover warning systems. When considering personality, drivers with low neuroticism had no strict requirements for time intervals. However, the extended time intervals were favorable for drivers with high neuroticism in developing SA. The present findings have reference implications for designers and engineers to set the time intervals of two-stage warning systems according to the neuroticism personality of drivers.

## Introduction

With the rapid development of automated technologies, future road driving will be largely reformed. Although automated driving has several bottlenecks to overcome, it can reduce crashes, prevent deaths and injuries, and alleviate traffic issues, such as congestion and emission (Anderson et al., 2014). The available automated driving (i.e., conditional automated driving, level 3; SAE International, 2018) no longer requires drivers to supervise the traffic environment. It allows them to engage in various non-driving-related tasks (NDRTs) during the automation. However, drivers are still expected to take over the vehicle when an automated system’s failure occurs (e.g., due to a stationary vehicle or obstacles in the current lane) (Zhang et al., 2019a) or when the driving automation system is about to leave its operational design domain (SAE International, 2018). The takeover process, which requires drivers to transfer from various NDRTs to driving tasks within a certain lead time, is of great importance for driving safety in level 3 (Society of Automotive Engineers, SAE) automated driving (McDonald et al., 2019).

## Related Work

### Preparation in the Takeover Process

With the increase of automated levels, drivers are more willing to engage in NDRTs (Jamson et al., 2013; De Winter et al., 2014). As a result, drivers are out of the loop when various NDRTs usually occupy their attention, cognitive resources, and motor resources (e.g., playing mobile games, watching videos, or taking a nap), to different extents (Jamson et al., 2013; Naujoks et al., 2017). Once hazards are detected, the automated driving system emits takeover warnings (i.e., takeover requests, TORs) to ask drivers to take over. As a response to TORs, drivers will experience a series of sub-processes for the takeover preparation, which includes the following: perceive takeover warnings, cognitively process the information, gain situation awareness (SA), make decisions, and resume motor readiness (i.e., repositioning their hands on the steering wheel and feet on the pedals) (Zeeb et al., 2015; Ito et al., 2016; Zhang et al., 2019a, b). All of these sub-processes are performed before taking actions.

### The Takeover Warning Systems

#### The Typical Takeover Warning Systems

Takeover warning systems have an important responsibility to connect “out-of-the-loop” drivers with dangerous traffic situations. The typical investigated takeover warning system was the single-stage warning system, which issues warning messages only once to drivers at a certain lead time. In the past few decades, researchers have dedicated to investigating the effectiveness of single-stage warning systems on takeover safety from its modality, lead time, interface, and so on (McDonald et al., 2019; see a review). For instance, Petermeijer et al. (2017) compared the effects of auditory, visual, and tactile takeover warnings on the takeover process in their study. They found that the warnings with auditory and tactile modalities can lead to drivers’ quicker responses than the warnings transmitted by visual modality. Wan and Wu (2018) prolonged the length of lead time and explored their effects on the takeover process. They found that the takeover request with lead time at 10–60 s led to safer takeover behaviors. Nevertheless, previous researchers mainly evaluated the effectiveness of takeover warning systems based on drivers’ resulting takeover performance (e.g., takeover time and acceleration indicators). Few studies concerned the effects of warning systems to the aspect of takeover preparation.

A good takeover preparation (e.g., gaining enough SA) before actual maneuvers is critical for performing steady and safe takeover actions. However, based on previous studies, we found that drivers often took over the vehicle without preparing sufficiently under the single-stage warning systems. Zhang et al. (2019b) had divided the total takeover response time into the perception response time and the movement response time (the indicator of motor readiness) in noncritical takeover conditions. They found that truck drivers spent approximately 4.61 s to achieve motor readiness when using the tablet during automated driving. Comparatively, obtaining SA required more cognitive resources and time for drivers, which includes the level of demand imposed on attentional resources by a situation, the supply of attentional resources in response to these situational demands, and the subsequent understanding of the situation (Taylor, 2017). In order to investigate the time that drivers require to build up situation awareness of a takeover situation, Lu et al. (2017) asked drivers to reproduce situations presented in videos with different lengths. The results suggested that drivers needed at least 7 s to estimate the basic topology of a situation. But substantial improvements in speed estimation were still achieved between 12- and 20-s videos. However, based on a review of 25 studies, Eriksson and Stanton (2017) found that drivers usually took over in 2.96 ± 1.96 s (including both preparation and taking actual actions) at the lead time range of 6.37 ± 5.36 s. This takeover reaction time was shorter than the necessary preparation time for drivers to perform motor preparation and gain SA, when they were all investigated under the single-stage warning systems. It indicated that drivers tended to take over rapidly without gaining enough SA and achieving good motor readiness under the single-stage warning systems.

#### Two-Stages Warning Systems and Time Intervals in the Takeover Process

The two-stage warning system, which warns drivers in two steps, is a promising method to guide drivers in achieving motor readiness and gaining sufficient SA. The first stage is designed to increase the drivers’ attention and shift their attention toward the situation without immediate actions. The second stage requires drivers to take actual maneuvers (Rhede et al., 2011; Werneke et al., 2014; Winkler et al., 2016). Some previous studies have investigated the effectiveness of the two-stage warning systems in the takeover process. For example, Ma et al. (2021) compared the effects of the single-stage and the two-stage warning systems on the takeover process. They found that the two-stage warning system can lead to better takeover performance, higher SA, less driving stress, and higher subjective ratings than the single-stage warning systems. Lu et al. (2019) compared the effect of TOR-only with the request, which provided a monitoring request (MR) (aims to let drivers achieve a monitoring transition) before a possible TOR (i.e., MR + TOR). They found that drivers spent a shorter takeover time and a longer minimum time to collision in the MR + TOR condition than in the TOR-only. These findings might result from a good preparation of drivers in the two-stage warning systems, which was formed during the time intervals between the first and the second warning stage.

Based on the effectiveness of the two-stage warning systems in the takeover (Ma et al., 2021), we raised the following question: to exert the advantages of this warning system to the maximum, what would be the appropriate length of time interval between the first and the second warning stage (i.e., the time interval in abbreviation in the whole manuscript) in the takeover process? Wogalter and Leonard (1999) suggested that a useful warning must hold attention for the time necessary to encode and store the message and prevent them from being distracted by other stimuli before the message is encoded satisfactorily. When the first warning stage is issued, the drivers’ attention can be captured. An extremely short time interval may not be sufficient for drivers to fully understand the situation and have good motor preparation for the takeover process. However, if the time interval between the first and second warning stage is too long, the drivers may be distracted by other stimuli and lose their attention again. Therefore, drivers may need optimal time intervals, while both the too short and too long time intervals degrade the takeover preparations and performance.

Although some previous studies had explored the sub-processes of a takeover, such as motor (physical) readiness, perception responses, and SA (Yoon et al., 2019; Lu et al., 2017; Zhang et al., 2019b), they had neither concerned how much time was necessary for drivers to make good preparation nor considered the possibility of a two-stage warning system in the takeover. Therefore, the influence of different time interval lengths on the drivers’ takeover process, especially the preparation process, remains unexplored.

### Personality and Its Interaction Effect With Time Intervals in the Takeover Process

Given that drivers have the critical responsibility of ensuring safety during the takeover, their takeover behaviors largely depend on themselves. Takeover behaviors may be influenced by driver-related factors such as personality, which refers to an internalized attribute of reasonable consistency and stability to which individual differences in behavior can be ascribed (Walters, 2000). Particularly, the neuroticism personality, which is defined as an inclination to experience negative emotions and difficulty in dealing with problems, had substantial effects on driving safety. Specifically, neuroticism was positively correlated with risky (Akbari et al., 2019) and aggressive driving behaviors (Zhang et al., 2017) as well as associated with the increased odds of road accidents (Alavi et al., 2017). The neuroticism personality was also an important driver characteristic to identify the drivers’ hazardous states (Darzi et al., 2018). Moreover, neuroticism affected the drivers’ SA. When testing the relationship between personality and SA during training in a navigation simulator, Saus et al. (2012) found that neuroticism was negatively associated with SA. It might be because drivers with high neuroticism generally generate more stress reactivity so that they were likely to divert their attention resources to worry about potential accidents and their performance when driving than do drivers with low neuroticism (Matthews and Desmond, 2001). As a result, the driving-related performance of drivers with high neuroticism is degraded.

As the takeover transition would also bring driving stress issues, we hypothesized that drivers with high neuroticism might behave poorly during the takeover process when compared to drivers with low neuroticism. Moreover, as the stress increased with perceived time constraints and unfamiliar driving conditions (Matthews et al., 1999; Scott-Parker et al., 2018), prolonging the available time might be an excellent way to alleviate the drivers’ stress, especially for drivers with high neuroticism. That is, drivers with high neuroticism would need relatively long time intervals in two-stage warning systems to alleviate their driving stress for takeover and become familiar with takeover situations. In this way, they can better utilize their cognitive resources to recover SA, make preparations, and take action rather than divert their attention resources to worry about other issues. However, the effect of neuroticism personality and its interaction effect with time intervals on the takeover process remain to be determined.

### Purpose and Hypotheses

Overall, the present study was driven by three purposes. The primary purpose was to explore the optimal time intervals of two-stage warning systems for the takeover process. We expected that there are optimal time intervals that can benefit the takeover preparation and the resulting performance. In contrast, excessively long time intervals (too long so that attention was distracted by other stimuli) or short time intervals (too short for making sufficient preparation) would degrade the takeover preparation and performance. Our study also examined whether the drivers’ neuroticism personality would influence the takeover process. We expected that drivers with high neuroticism would have inadequate preparation as well as a bad takeover performance. Moreover, the interaction effect between time intervals and neuroticism was investigated. We hypothesized that drivers with high neuroticism needed longer lengths of time intervals than do drivers with low neuroticism.

## Materials and Methods

### Participants

A total of 32 participants (19 males and 13 females) with ages that ranged from 19 to 26 years (*M* = 23.0 years, SE = 0.3), who were recruited from Zhejiang Sci-Tech University, participated in our experiment. These participants had a valid driving license and normal or corrected-to-normal vision. Their average number of years of driving was 2.7 years (SE = 0.3) and the average driving experience in the past year was 205.7 km (SE = 62.9). No driver had previously experienced conditional automated driving.

### Experiment Design and Measures

This study adopted a 2 × 4 mixed design, with neuroticism as the between-subjects factor (high/low neuroticism) and time interval as the within-subjects factor (3, 5, 7, and 9 s). Participants were classified into two groups according to their neuroticism scores by the median split method (i.e., low neuroticism group: below and equal to the median; high neuroticism group: above the median) (Tement et al., 2020). Subsequently, each participant completed four takeovers that corresponded to different time intervals. The sequence of the time intervals was balanced by the Latin square.

#### Neuroticism Personality

We used the Neuroticism–Anxiety subscale of the Chinese version of the Zuckerman–Kuhlman Personality Questionnaire to measure the drivers’ neuroticism personality (ZKPQ-50; Zuckerman et al., 1993; Wu et al., 2000). The subscale includes 19 items that measure emotional upset, tension, worry, fearfulness, obsessive indecision, lack of self-confidence, and sensitivity to criticism. The internal reliability (Cronbach’s alpha coefficient) of this subscale was 0.81. Besides, 10 items of a dissimulation (infrequency or lie) scale were randomly inserted into the Neuroticism–Anxiety subscale to assess the validity of individual records because it is related to neuroticism personality (Furnham et al., 1998). Participants decided whether they agree or disagree with the described statement items. They received scores if their answers meet the characteristics of neuroticism. A critical criterion for data selection was the score of under 3 in the dissimulation scale (Wu et al., 2000).

#### Takeover Performance

Three measures were extracted to evaluate the drivers’ takeover performance. (1) hands-on-steering-wheel time (in seconds) is an indicator of motor readiness, which was measured from the moment the warning (the first warning in our study) was issued until the participants placed at least one hand on the steering wheel (Lu et al., 2019); (2) takeover time (in seconds) is the minimum time between the issuance of the warning (the second warning in our study) and when the steering angle is greater than 2° or the brake percentage exceeded 10% (Gold et al., 2013); (3) maximum resulting acceleration (in meters per square second) is the takeover quality indicator, which was defined as:

m⁢a⁢x⁢i⁢m⁢u⁢m⁢a⁢c⁢c⁢e⁢l⁢e⁢r⁢a⁢t⁢i⁢o⁢nresulting=maximun⁢accelerationlongitudinal+2accelerationlateral2

This indicator was collected from the onset of the second warning to the moment when the drivers overtook the position of the takeover hazards. A higher acceleration suggested less safe reactions to warnings (Hergeth et al., 2017).

#### Situation Awareness

During the takeover, the drivers’ SA was measured by the Situation Awareness Rating Technique (SART), which was conducted after each trial to avoid interrupting the takeover process (Selcon and Taylor, 1990; Nguyen et al., 2019). The SART questionnaire contained 10 questions for three dimensions, namely, demand from attentional resources (D), the supply of attentional resources (S), and understanding of the situation (U). Drivers evaluated each question based on a seven-point Likert scale depending on the takeover situation they just experienced. Finally, the SA score was calculated as the sum of U and S less D.

#### Appropriateness and Usefulness of the Time Intervals

A 15-point rating scale was used to evaluate the appropriateness and usefulness of the time intervals that the participants perceived (Heller, 1981; Appendix). Participants first selected one out of five categories (appropriateness: far too long, too long, just right, too short, and far too short; usefulness: very useless, useless, moderate, useful, and very useful) and then narrowed down their answer using three subcategories (−, 0, +). The appropriateness and usefulness results of the time intervals were first transformed into scores that range from 1 to 15 and then averaged. For appropriateness, “8” represented the most appropriate rating, which corresponded to “just right—0”; with the decrement or increment of this rating, time intervals were deemed longer or shorter, respectively.

### Apparatus

**FIGURE 1:**
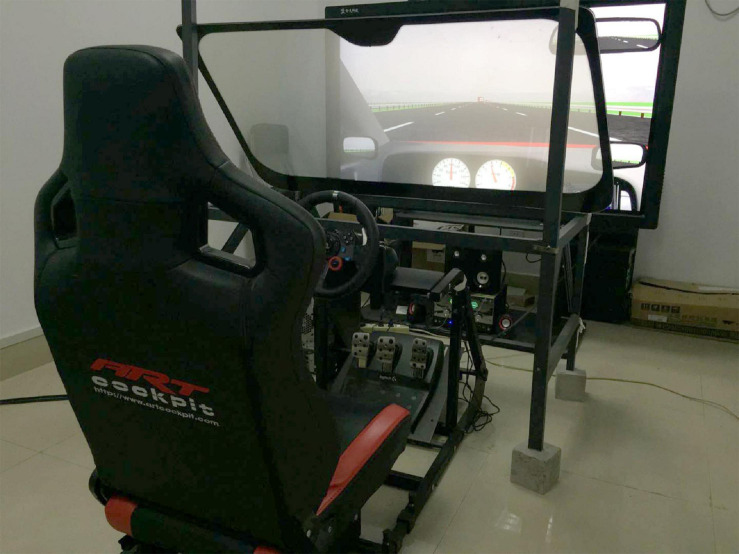
Driving simulation in the study.

A fixed-based driving simulator was used in the present study (see Figure 1). The driving simulator consisted of a driving simulator software (STISIMDRIVE M100K, Systems Technology Inc., Hawthorne, CA, United States), a ThinkCentre [Precision M6600t, Intel Core (TM) i5-6500 CPU 3.20 GHz], an adjustable seat, and an operating system (Logitech MOMO, Newark, CA, United States) that included a steering wheel, an accelerator, and a brake pedal. A 60-in. viewing screen with 1,920 × 1,080 pixel resolution was placed in front of the operating system to display the driving scenarios. A loudspeaker was placed below the screen to emit scenario sounds and warning messages. During the whole experiment, a Logitech C270 webcam with 1,280 × 720 pixel resolution was used to record the drivers’ behaviors. All driving activities were recorded by using the simulated software at a frequency of 120 Hz.

### Driving Scenarios

Driving scenarios were built by the simulator software. On a six-lane highway (three lanes in each direction), the automated system drove the vehicle in the middle lane at a speed of 100 km/h. Four hazards concerning lane changing were included: (1) a broken-down truck, (2) obstacles, (3) a traffic accident, and (4) a work zone. All hazards are briefly described in Table 1. The four hazards would occupy the front-and-left or front-and-right lanes (randomly arranged), thereby leaving only one lane for the drivers to pass. Moreover, these hazards already happened before being detected by the ego vehicle, and no other vehicles existed during the takeover. Thus, the takeover events were non-urgent in general. The sequence of hazards was counterbalanced with four time intervals.

**TABLE 1:** Takeover scenarios.

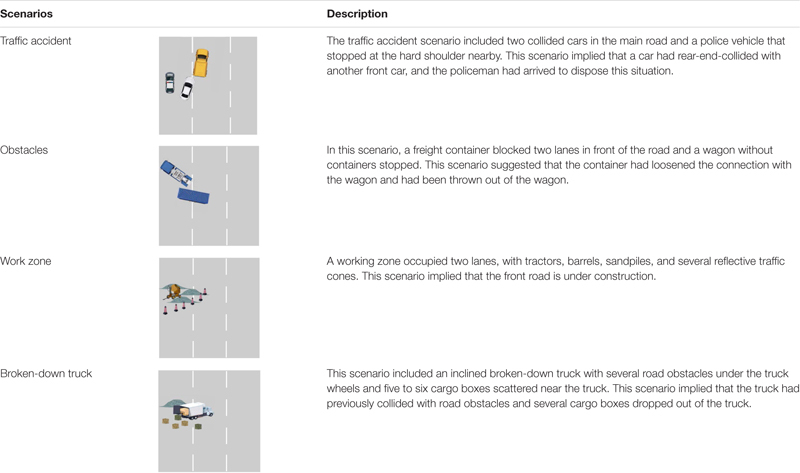

### Two-Stages Warning Systems

The auditory modality was used to provide warning messages to drivers for omnidirectional advantage (Bazilinskyy and de Winter, 2015) and effectiveness in the takeover performance (Petermeijer et al., 2017). The two-stage warning comprised two short auditory messages. These messages were emitted at different lead times, which refer to the time-to-collision between the automated vehicle and the critical hazard (e.g., a stationary vehicle) if the automated vehicle continues at its current speed and current path at the onset of a TOR (Lee, 1976; McDonald et al., 2019). When approaching the takeover hazards, a short warning message, “attention please,” was firstly issued at the lead times of 10, 12, 14, and 16 s. Subsequently, the second warning message, “take over please,” was displayed at the lead time of 7 s, which was the most commonly used lead time (McDonald et al., 2019); this phenomenon leaves the time intervals between the first and the second warning for 3, 5, 7, and 9 s, respectively. Both messages had a speech rate of 150 words/min, a loudness of 70 dB, and a duration of 1,100 ms.

### Non-driving-Related Tasks

During automated driving, the participants were required to play the game Tetris installed previously on a smartphone. The difficulty level of Tetris was consistent for each participant (i.e., the falling speed of pieces is 1.6 square/s). Participants were instructed to immerse themselves in Tetris and ensure the takeover safety once the takeover warning was issued. To control the effect of motivation, they were told that they could receive scores based on a composite of their takeover performance (weight, 60%) and Tetris game scores (weight, 40%); the top three participants would receive rewards of 50, 75, and 100 yuan, respectively.

### Procedures

After being welcomed, participants were notified to complete the neuroticism subscale, in which 10 dissimulation items were randomly inserted (approximately 5 min). Then, the experimenter decided whether the participant can join in our experiment according to their score of dissimulation. A total of 39 participants completed the scale, and 32 of them met the requirements to join the simulated experiment.

The chosen participants were instructed to complete the experiment. Firstly, they signed informed consent forms and filled out the demographic questionnaires related to their age, gender, driving experience (manual driving and automated or takeover driving), and health state. Next, the participants were instructed to familiarize themselves with the driving simulator (the feeling of the steering wheel and the tightness of pedals) through manual driving on a highway road (5 min). Then, they practiced the takeover process twice (10 min). Scenarios in the practice trial were designed similarly, to one of the scenarios used in the formal trial. All participants played Tetris on a smartphone during the automated driving for either 3 or 4 min, which were randomly arranged with four takeover hazards to avoid the anticipation effect. The participants were instructed to pay attention to the driving scenarios when they hear the first warning “attention please” and take over the ego vehicle when they hear the second warning “take over please.” Participants were told that after the second warning is issued, they can turn off the automated system by steering the wheel or pressing the brake pedal. When completing the takeover, the participants drove manually for 1 min and the program ended automatically. In the formal test, each participant finished four trials. At the end of each trial, they evaluated the appropriateness and usefulness of the time intervals they just received and their understanding of the takeover hazards based on SART. All participants were required to report once they experience any typical simulator sickness symptoms (e.g., feel dizzy, have a headache, want to vomit, etc.) during the experiment. No simulator sickness was reported. The whole experimental process lasted for approximately 60 min, and each participant was compensated with 30 yuan for joining the experiment. After all the participants completed the experiment, the top three participants received rewards for 50, 75, and 100 yuan, respectively.

### Data Analysis

IBM SPSS Statistics 26.0 was used to analyze the data. Firstly, we checked the normal distribution of all dependent variables using the Kolmogorov–Smirnov test. Then, we conducted log transformation for data that did not follow normal distribution. Next, we adopted the linear mixed model (LMM), which consisted of fixed effects and random effects, to conduct the analysis. Relative to ANOVA, LMM has the advantage of eliminating the influence of sequence effects on dependent variables by viewing it as a random effect (Baayen et al., 2008). In this study, neuroticism, time intervals, and interaction were considered as the fixed effects. The sequence of tests, direction of hazards, time length of automated driving, and driving experience (license year and the past year driving) were considered the random effects. The least significant difference (LSD) method was adopted for *post hoc* analysis. The significance level was set to 0.05.

## Results

The present study conducted a driving-simulated experiment to investigate the effect of time intervals of two-stage warning systems and neuroticism personality on the takeover process. In the present study, all participants successfully took over the ego vehicle without collisions (100% accident avoidance rate). The mean and standard errors (SEs) of all dependent variables for time intervals and neuroticism are listed in Tables 2, 3, respectively.

**TABLE 2:** Mean and standard errors of the takeover performance and subjective ratings with different time intervals.

Measures	3 s	5 s	7 s	9 s	*F*	*p*
***Takeover performance***						
Hands-on-steering-wheel time (s)	3.2967 (0.163)	3.636 (0.267)	4.374 (0.401)	5.394 (0.601)	4.942	<0.01**
Takeover time (s)	1.519 (0.082)	1.154 (0.076)	1.252 (0.091)	1.516 (0.104)	17.519	<0.001***
Maximum resulting acceleration (m/s^2^)	1.534 (0.150)	1.624 (0.197)	1.480 (0.135)	1.607 (0.153)	0.340	0.797
***Subjective measures***						
Situation awareness	23.719 (1.448)	26.469 (1.326)	25.531 (1.354)	25.906 (1.410)	2.792	<0.05*
Appropriateness	9.656 (0.369)	7.500 (0.301)	7.125 (0.353)	4.938 (0.419)	46.930	<0.001***
Usefulness	10.500 (0.428)	11.844 (0.305)	11.688 (0.493)	10.250 (0.428)	4.754	<0.05*

Values shown as mean (SE). *p < 0.05, **p < 0.01, ***p < 0.001.

**TABLE 3:** Mean and standard errors of the takeover performance and subjective ratings for low-neuroticism and high-neuroticism drivers.

Measures	Low neuroticism	High neuroticism	*F*	*p*
***Takeover performance***				
Hands-on-steering-wheel time (s)	4.558 (0.293)	3.767 (0.293)	3.310	0.076^+^
Takeover time (s)	1.468 (0.071)	1.253 (0.056)	2.477	0.125
Maximum resulting acceleration (m/s^2^)	1.346 (0.144)	1.773 (0.144)	2.023	0.165
***Subjective measures***				
Situation awareness	27.422 (0.905)	23.391 (0.987)	3.678	0.065^+^
Appropriateness	6.813 (0.333)	7.797 (0.316)	4.783	<0.05*
Usefulness	10.500 (0.220)	11.641 (0.360)	10.528	<0.05*

Values shown as mean (SE). 0.05 < ^+^p < 0.1, *p < 0.05, **p < 0.01, ***p < 0.001.

### Hands-On-Steering-Wheel Time

**FIGURE 2:**
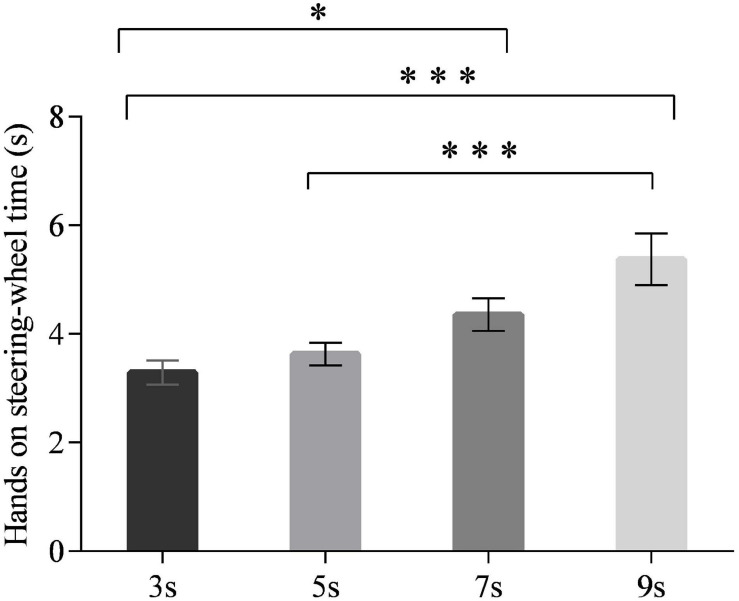
Hands on steering-wheel time for different time intervals. (Notes. *p < 0.05, ***p < 0.001).

The main effect of time intervals on the hands-on-steering-wheel time was significant [*F*_(3,90)_ = 4.942, *p* < 0.01] (Figure 2). The drivers’ hands-on-steering-wheel time increased with the time intervals. The *post hoc* test revealed that drivers were quicker to put hands on the steering wheel under the 3-s time interval than under the 7-s (*p* < 0.05) and 9-s (*p* < 0.001) time intervals, respectively. They also responded more swiftly by putting their hands on the steering wheel under the 5-s time interval than the 9-s time interval (all *p* < 0.001). The main effect of neuroticism on the hands-on-steering-wheel time was marginally significant [*F*_(1,30)_ = 3.310, *p* = 0.076]. Compared with the drivers with low neuroticism, the drivers with high neuroticism responded slightly quicker in putting their hands on the wheel (*p* = 0.076). No interaction effect was observed [*F*_(3,90)_ = 0.516, *p* = 0.673].

### Takeover Time

**FIGURE 3:**
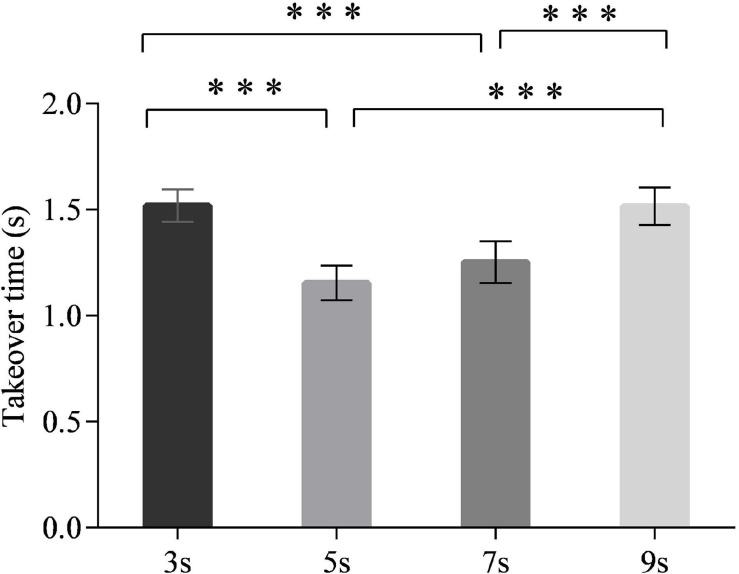
Takeover time for different time intervals. (Notes. ***p < 0.001).

**FIGURE 4:**
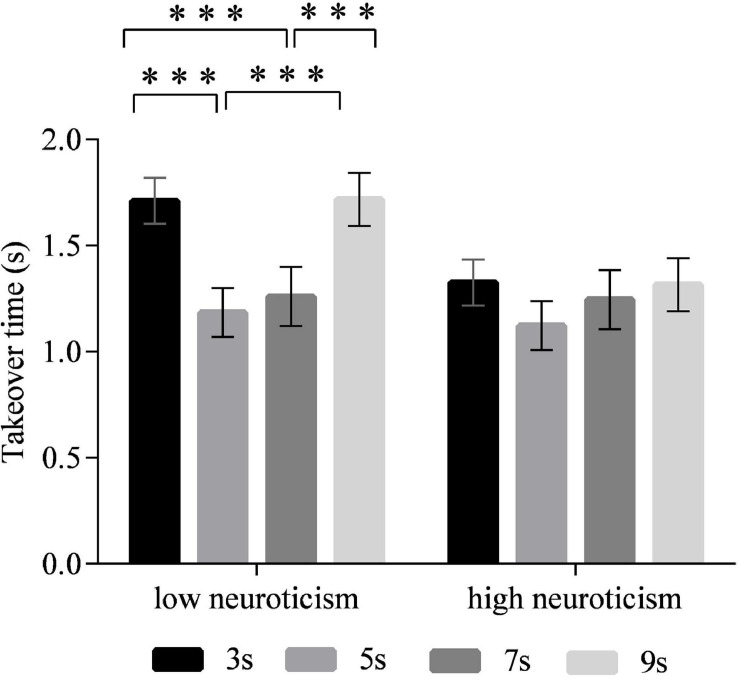
Takeover time of different time intervals for drivers with the low and high neuroticism. (Notes. ***p < 0.001).

**FIGURE 5:**
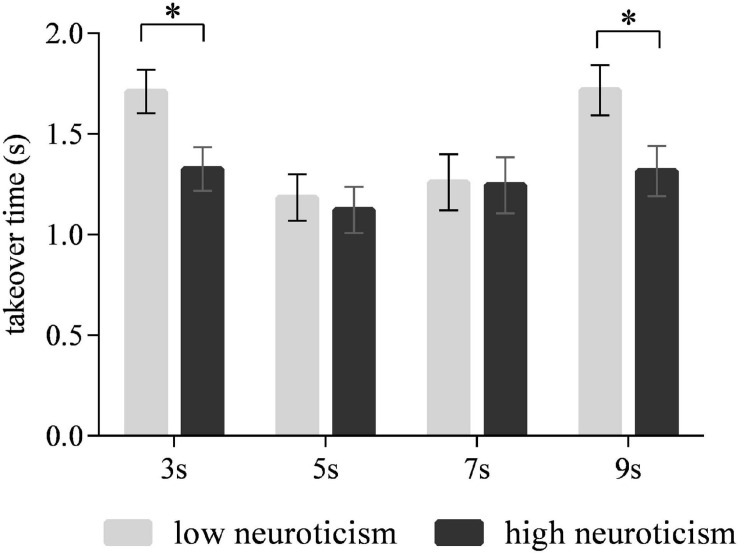
Takeover time for drivers with low and high neuroticism under different time intervals. (Notes. *p < 0.05).

The main effect of time intervals on the takeover time was significant [*F*_(3,90)_ = 17.519, *p* < 0.001] (Figure 3). The *post hoc* test revealed that the drivers took over the ego vehicle significantly faster in the 5-s time interval than in the 3- and 9-s time intervals (*p* < 0.001 for each comparison). Also, the 7-s time interval led to shorter takeover times than the 3- and 9-s time intervals (*p* < 0.001 for each comparison). No significant differences in the takeover time were observed between the 5- and 7-s time intervals. The interaction effect of time intervals and neuroticism on the takeover time was significant [*F*_(3,90)_ = 3.184, *p* < 0.05] (Figure 4). The simple effect analysis revealed that for the drivers with low neuroticism, the takeover time under the 5- and 7-s time intervals was significantly shorter than those under the 3- and 9-s time intervals (*p* < 0.001 for each comparison). However, the four time intervals led to no significant difference in the takeover time for drivers with high neuroticism. Moreover, under both the 3- and 9-s time intervals, the drivers with high neuroticism had longer takeover times than the drivers with low neuroticism (*p* < 0.05). In comparison, there were no significant differences between two driver groups under the 5- and 7-s time intervals (Figure 5). The main effect of neuroticism on the takeover time was not significant [*F*_(1,30)_ = 2.477, *p* = 0.125].

### Maximum Resulting Acceleration

No main significant effect of time interval [*F*_(3,90)_ = 0.340, *p* = 0.797] or neuroticism [*F*_(1,30)_ = 2.023, *p* = 0.165] or the interaction effect of two factors [*F*_(3,90)_ = 0.167, *p* = 0.918] was observed on maximum resulting acceleration.

### Situation Awareness

**FIGURE 6:**
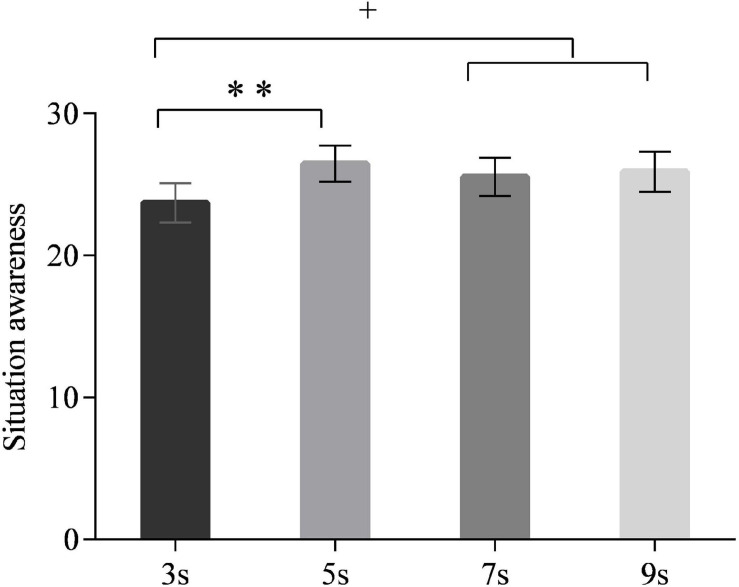
Situation awareness for different time intervals. (Notes. **p < 0.01, 0.05 < ^+^*p* < 0.1).

**FIGURE 7:**
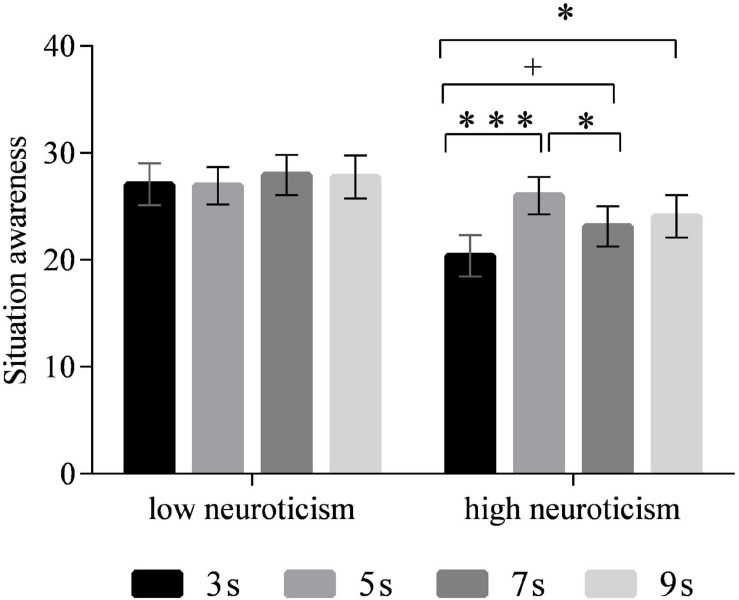
Situation awareness of different time intervals for drivers with the low and high neuroticism. (Notes. 0.05 < ^+^*p* < 0.1, *p < 0.05, ***p < 0.001).

**FIGURE 8:**
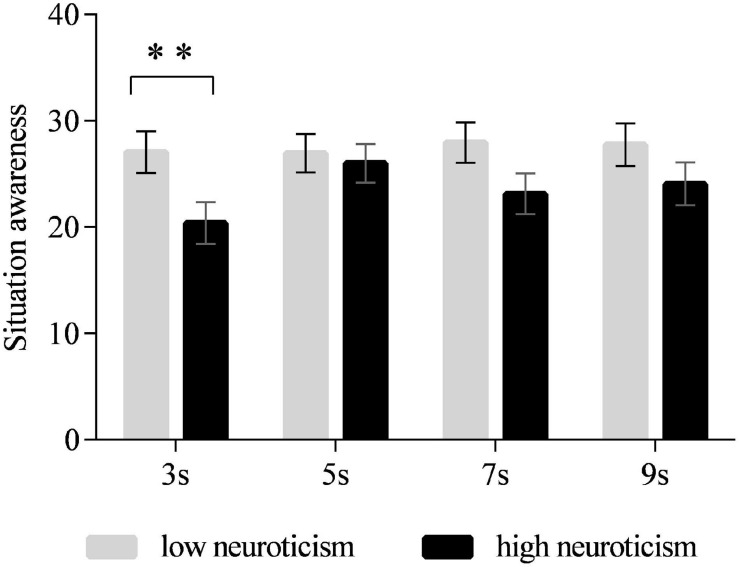
Situation awareness for drivers with low and high neuroticism under different time intervals. (Notes. **p < 0.01).

A main significant effect of time interval on SA was observed [*F*_(3,90)_ = 2.792, *p* < 0.05]. The *post hoc* test revealed that the drivers’ SA in the 3-s time interval was lower than those in 5 s (all *p* < 0.01), 7 s (marginally significant, all *p* = 0.074), and 9 s (marginally significant, all *p* = 0.061) (see Figure 6). No significant differences were observed between 5, 7, and 9 s. The main effect of neuroticism was marginally significant [*F*_(1,30)_ = 3.678, *p* = 0.065]. High-neuroticism drivers tended to have lower SA than the low-neuroticism drivers (*p* = 0.065). The interaction effect of time intervals and neuroticism was also marginally significant [*F*_(3,90)_ = 2.734, *p* = 0.059] (Figure 7). The simple effect analysis revealed that for the high-neuroticism drivers, the 3-s time interval led to lower SA than the 5 s (all *p* < 0.001), 7 s (all *p* = 0.066), and 9 s (all *p* < 0.05), and the 5-s time interval led to higher SA than the 7-s time interval (all *p* < 0.05). However, no significant differences in SA were observed between the four time intervals for drivers with low neuroticism. Moreover, when the time interval was 3 s, drivers with low neuroticism had significantly higher SA than the drivers with high neuroticism (*p* < 0.01) while this effect was not observed under the other time intervals (Figure 8).

## Appropriateness of Time Intervals

**FIGURE 9:**
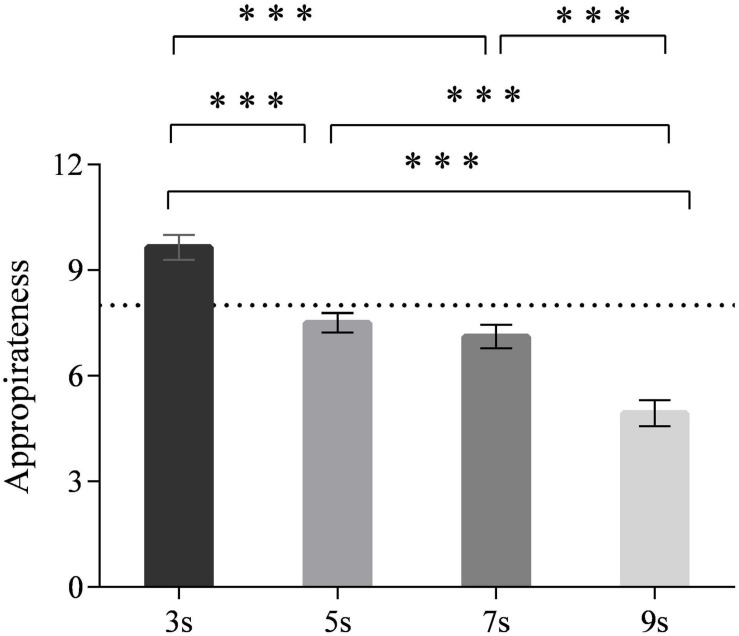
Appropriateness ratings for different time intervals. (Notes. ***p < 0.001).

A significant main effect of time intervals [*F*_(3,90)_ = 46.930, *p* < 0.001] on appropriateness was observed (Figure 9). Both the 5- and 7-s time intervals were rated appropriate, whereas the 3-s time interval was rated too short and the 9-s time interval rated too long. The comparisons of each pair of time intervals were significant at the 0.001 level, except for the 5 and 7 s. The main effect of neuroticism was also significant [*F*_(1,30)_ = 4.783, all *p* < 0.05]. Highly neurotic drivers tended to rate time intervals more appropriate in general than did drivers with low neuroticism, who reckoned that the time intervals they received were relatively long (all *p* < 0.05). No interaction effect was found [*F*_(3,90)_ = 1.497, *p* = 0.216].

### Usefulness of Time Intervals

**FIGURE 10:**
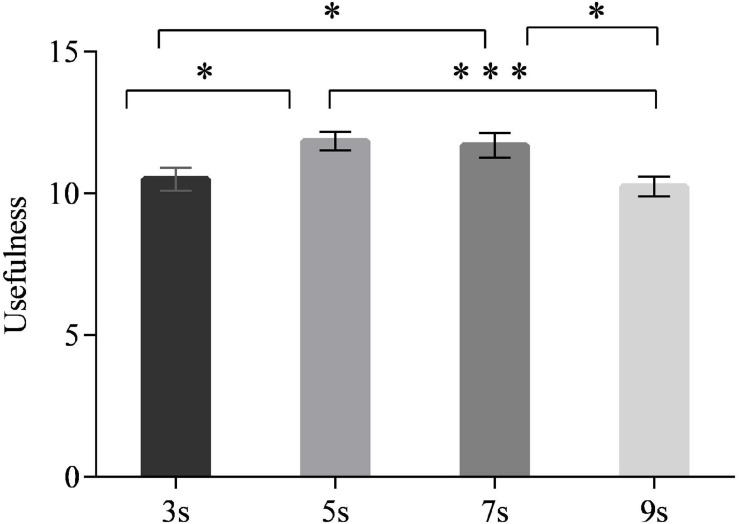
Usefulness ratings for different time intervals. (Notes. *p < 0.05, ***p < 0.001).

Both main effects of time intervals [*F*_(3,90)_ = 4.754, *p* < 0.05] and neuroticism [*F*_(1,30)_ = 10.528, *p* < 0.05] on usefulness were significant. As Figure 10 shows, time intervals of 5 and 7 s were rated more useful than 3 and 9 s (*p* < 0.05 for each comparison, except for the comparison between 5 and 9 s, all *p* < 0.001). The drivers with high neuroticism rated time intervals as more useful in general than those with low neuroticism (all *p* = 0.015). No interaction effect was observed [*F*_(3,90)_ = 0.950, *p* = 0.448].

## Discussion

The present study explored the effect of time intervals and the neuroticism personality of drivers on the takeover process. Firstly, the results revealed that the 5-s time interval could result in fast responses in achieving motor readiness and sufficient SA for drivers. Secondly, both 5- and 7-s time intervals can lead to more rapid takeover responses and were rated more appropriate and useful than the 3- and 9-s time intervals. Thirdly, we found that drivers with high neuroticism tended to take over immediately as soon as they receive the takeover messages, regardless of whether the time intervals were enough to make adequate preparations, at the cost of SA deficiency.

### Effect of Time Intervals on the Takeover Process

The results suggested that 5 s was a good time interval for fast motor readiness preparation and obtaining good SA. Firstly, we found that the drivers’ reaction times in placing hands on the steering wheel increased with time intervals. Notably, no significant differences on the hands-on-steering-wheel time were observed between the 3- and 5-s time intervals. That is, both time intervals led to fast motor readiness. Previous researchers suggested that drivers respond more quickly to more critical situations (Naujoks et al., 2014). It might be because the takeover situations in both the 3- and 5-s time intervals were perceived more critical by drivers so that they were generally faster to place their hands on the steering wheel in these circumstances. Nevertheless, although drivers, in the 3-s time interval, made similarly, fast motor readiness to that in the 5-s time interval, their SA in the 3-s time interval was the least, lower than those in the 5-, 7-, and 9-s time intervals. Moreover, no significant differences were observed between the 5-, 7-, and 9-s time intervals. This finding further indicated that the time interval should be at least 5 s for drivers to gain the same SA level as that in the longer time intervals. This time was shorter than that in the previous finding, indicating that drivers needed at least 7 s to gain enough SA (Lu et al., 2017). It could be because the takeover scenarios in the present experiment had no surrounding vehicle while those of Lu et al. (2017) had; thereby, the takeover scenarios in the present experiment were comparatively simple. Besides, the drivers can keep gaining SA after the second warning was issued. Thus, in our study, the time interval (i.e., 5 s) was sufficient for drivers to gain a good SA level.

Moreover, a U-shaped relationship between the time intervals and the takeover time was observed. Specifically, drivers took over faster in the 5- and 7-s time intervals than they did in the 3- and 9-s time intervals. This finding conformed to our hypothesis, which expected proper time intervals rather than the too short or too long time intervals for better takeover preparations and performance. The reason may be that the drivers’ SA was relatively low in the shorter (i.e., 3 s) time interval. Thus, more time was needed for drivers to interpret scenarios and take responses. For the longer time interval, 9 s may be too long, and thereby the drivers were distracted by other issues (Brown et al., 2001; Winkler et al., 2016; Wan and Wu, 2018); once the second warning was emitted, the drivers needed more time to fix their attention again. Therefore, the mid-length time intervals (i.e., 5 and 7 s) were optimal for drivers to gain enough SA and avoid being distracted by other information. Furthermore, drivers’ subjective ratings also supported the 5- and 7-s time intervals. Both 5 and 7 s were rated as the most appropriate time intervals for the drivers to make preparations, whereas the 3-s time interval was perceived insufficient and the 9-s time interval was perceived slightly redundant. Also, 5 and 7 s were rated more useful by drivers than were the 3- and 9-s time intervals. Nevertheless, the results suggested that the drivers’ accelerations in the takeover were not varied according to time intervals. As the takeover transition in this study was non-urgent in terms of the noncritical takeover scenarios and adequate lead time (i.e., 7 s) of the second warning stage, the short time interval of 3 s was sufficient for them to exert a smooth takeover acceleration. Thus, the resulting acceleration would not be improved when increasing the time intervals.

### Effect of Neuroticism on the Takeover Process

The present study also concerned how the “neuroticism” personality of drivers influenced the takeover process. Firstly, although drivers with high neuroticism responded quickly in making motor readiness, they exerted lower SA compared with the drivers with low neuroticism. The results suggested that drivers with high neuroticism were quicker to place their hands on the steering wheel than were drivers with low neuroticism. The reason may be the production of stress reactivity related to neuroticism personality during the takeover. Drivers with high neuroticism were more likely to appraise situations as threatening and challenging, thereby intensifying their stress reactivity (Schneider, 2004; Saus et al., 2012). As a result, this stress reactivity led to faster responses (Colemere, 1998). Nevertheless, even though the drivers with high neuroticism achieved motor readiness swiftly, they tended to gain lower SA than the drivers with low neuroticism. Considering that individuals with high neuroticism had difficulty in controlling negative emotions, they were likely to divert their attention to personal concerns rather than the current driving environment, which would decrease cognitive capability (Matthews and Desmond, 2001; Tement et al., 2020). As a result, their gain of SA, which also requires cognitive resources, was interfered.

Moreover, we found that drivers with high neuroticism also tended to rate time intervals as more appropriate and useful than did drivers with low neuroticism. As mentioned before, more stress reactivity was generated by highly neurotic drivers, and Jovanović et al. (2011) found that individuals with high neuroticism are inefficient in their attempts to overcome this stress. When provided with time intervals, the stress state of highly neurotic drivers might be significantly alleviated, explaining why these drivers were more satisfied with time intervals in general.

### Interaction Effect on the Takeover Process

Interaction effects between neuroticism and time intervals were observed with the takeover time and SA. Drivers with high neuroticism took over rapidly in all the time intervals, whereas drivers with low neuroticism took over faster in the 5- and 7-s time intervals than they did in the 3- and 9-s time intervals. Based on these findings, it seems that highly neurotic drivers were not susceptible to different time intervals in general. Nevertheless, the SA of drivers with high neuroticism under the 3-s time interval was significantly lower than those in the 5-, 7-, and 9-s time intervals. Their SA under the 5-s time interval was higher than that at 7 s. It suggested that these drivers needed a 5-s time interval to gain sufficient SA. Although drivers with low neuroticism responded more slowly in the 3- and 9-s time intervals, they gained the same SA levels in the 3- and 9-s time intervals as those in the 5- and 7-s time intervals. These results suggested that drivers with high neuroticism responded rapidly merely according to warning instructions, regardless of the time intervals. When the time interval was relatively short, they failed to adjust themselves to gain sufficient SA. However, drivers with low neuroticism responded by judging whether they gained enough SA. When they came across the disadvantageous time intervals, they adjusted themselves by responding more slowly for the sake of gaining sufficient SA. Therefore, highly neurotic drivers had fewer safety coping strategies than did drivers with low neuroticism, and relatively long time intervals were suitable for those drivers to obtain a good understanding of the takeover situations.

To summarize, these findings have reference implications for designers and engineers to set time intervals for two-stage warning systems in vehicles. Considering drivers’ personality, it is suggested to provide the highly neurotic drivers with a longer time interval to develop their SA.

### Limitations and Future Work

Several limitations existed in this study. Firstly, we selected a post-trial subjective technique (i.e., SART) to measure the drivers’ SA in order to not interfere with the task execution. In this way, however, we actually measured the SA of the whole takeover process, although the SA of the time intervals can be reflected from it. Moreover, the post-trial rated SA may be influenced by the drivers’ recalling ability (Nguyen et al., 2019), and it may correspond to performance (Endsley, 1995). Thus, multiple SA measures such as freeze probe techniques, read time probe techniques, observer rating techniques, and performance measures should be considered in future studies (Nguyen et al., 2019). Secondly, the takeover scenarios were non-urgent (events already happened) and no other road elements existed (e.g., vehicles or curves) during the takeover process. In order to enhance the ecological validity of the experiment, a diverse pool of takeover events such as dynamic traffic situations and various road types should be considered in future studies. Thirdly, the participants in our study lacked driving experience, which can influence the gain of SA during the takeover (Wright et al., 2016). To generalize the present findings to most drivers, future studies should include more experienced drivers. Fourthly, the present study was conducted in driving simulators. Although validation of simulated driving systems in testing driving ability has been confirmed (Gibbons et al., 2014), future studies should consider conducting this experiment on a real highway to produce more realistic results.

## Conclusion

This study investigated the effect of time intervals in two-stage warning systems and the neuroticism personality of drivers on the takeover process. Among all time intervals, 5 s was the optimal time interval for drivers to make preparations and take over in general. When considering personality, drivers with low neuroticism had no strict requirement for time intervals. However, it is suggested to provide highly neurotic drivers with more extended time intervals to develop SA. The present findings have reference implications for designers and engineers to consider different time intervals for two-stage warning systems according to drivers’ personality.

## Data Availability Statement

The raw data supporting the conclusion of this article will be made available by the authors, without undue reservation.

## Ethics Statement

The studies involving human participants were reviewed and approved by Institutional Review Boards of Zhejiang Sci-Tech University. The patients/participants provided their written informed consent to participate in this study.

## Author Contributions

SM: conceptualization, methodology, writing – review and editing, and funding acquisition. HL: supervision, project administration, and funding acquisition. WZ: conceptualization, methodology, software, validation, formal analysis, investigation, resources, writing – original draft, and visualization. YZ: data curation, formal analysis, investigation, and resources. ZY: conceptualization, methodology, and funding acquisition. CK: conceptualization and supervision. CW: supervision and writing – review and editing. JS: software and visualization. All authors contributed to the article and approved the submitted version.

## Conflict of Interest

The authors declare that the research was conducted in the absence of any commercial or financial relationships that could be construed as a potential conflict of interest.

## Appendix

### The Appropriateness and Usefulness Rating Scale

**Table:** How appropriate was the time interval just experienced?

Far too long			Too long			Just right			Too short			Far too short		
**–**	**0**	**+**	**–**	**0**	**+**	**–**	**0**	**+**	**–**	**0**	**+**	**–**	**0**	**+**

How useful was the time interval just experienced?														

**Very useless**			**Useless**			**Moderate**			**Useful**			**Very useful**		

**–**	**0**	**+**	**–**	**0**	**+**	**–**	**0**	**+**	**-**	**0**	**+**	**–**	**0**	**+**
